# Cinacalcet corrects hypercalcemia in mice with an inactivating G**α**_11_ mutation

**DOI:** 10.1172/jci.insight.96540

**Published:** 2017-10-19

**Authors:** Sarah A. Howles, Fadil M. Hannan, Caroline M. Gorvin, Sian E. Piret, Anju Paudyal, Michelle Stewart, Tertius A. Hough, M. Andrew Nesbit, Sara Wells, Stephen D.M. Brown, Roger D. Cox, Rajesh V. Thakker

**Affiliations:** 1 Academic Endocrine Unit, Radcliffe Department of Medicine, University of Oxford, Oxford, United Kingdom.; 2 Department of Musculoskeletal Biology, Institute of Ageing and Chronic Disease, University of Liverpool, Liverpool, United Kingdom.; 3 Mammalian Genetics Unit and Mary Lyon Centre, Medical Research Council (MRC) Harwell Institute, Harwell Science and Innovation Campus, United Kingdom.; 4 Biomedical Sciences Research Institute, Ulster University, Coleraine, United Kingdom.

**Keywords:** Endocrinology, Metabolism, Calcium, Drug therapy, G-proteins

## Abstract

Loss-of-function mutations of *GNA11*, which encodes G-protein subunit α_11_ (Gα_11_), a signaling partner for the calcium-sensing receptor (CaSR), result in familial hypocalciuric hypercalcemia type 2 (FHH2). FHH2 is characterized by hypercalcemia, inappropriately normal or raised parathyroid hormone (PTH) concentrations, and normal or low urinary calcium excretion. A mouse model for FHH2 that would facilitate investigations of the in vivo role of Gα_11_ and the evaluation of calcimimetic drugs, which are CaSR allosteric activators, is not available. We therefore screened DNA from > 10,000 mice treated with the chemical mutagen *N*-ethyl-*N*-nitrosourea (ENU) for *GNA11* mutations and identified a Gα_11_ variant, Asp195Gly (D195G), which downregulated CaSR-mediated intracellular calcium signaling in vitro, consistent with it being a loss-of-function mutation. Treatment with the calcimimetic cinacalcet rectified these signaling responses. In vivo studies showed mutant heterozygous (*Gna11^+/195G^*) and homozygous (*Gna11^195G/195G^*) mice to be hypercalcemic with normal or increased plasma PTH concentrations and normal urinary calcium excretion. Cinacalcet (30mg/kg orally) significantly reduced plasma albumin–adjusted calcium and PTH concentrations in *Gna11^+/195G^* and *Gna11^195G/195G^* mice. Thus, our studies have established a mouse model with a germline loss-of-function Gα_11_ mutation that is representative for FHH2 in humans and demonstrated that cinacalcet can correct the associated abnormalities of plasma calcium and PTH.

## Abstract 2

Cinacalcet corrects hypercalcemia in a mouse model for familial hypocalciuric hypercalcemia type 2 (FHH2) caused by a germline loss-of-function G-protein subunit α11 (Gα11) mutation, Asp195Gly.

## Introduction

Familial hypocalciuric hypercalcemia (FHH) is an autosomal dominant disorder of extracellular calcium (Ca^2+^_o_) homeostasis characterized by lifelong elevations in serum calcium concentrations in association with normal or mildly elevated serum parathyroid hormone (PTH) concentrations and normal or low fractional excretion of calcium (1–4). FHH is caused by a reduction in the sensitivity of the Ca^2+^_o_-sensing receptor (CaSR) signaling pathway to alterations in the prevailing Ca^2+^_o_ concentration ([Ca^2+^]_o_) (1–4). The CaSR is a widely expressed family C GPCR that regulates PTH secretion and urinary calcium excretion by transducing elevations in [Ca^2+^]_o_ into multiple intracellular signaling cascades in the parathyroid glands and kidneys, respectively (5, 6). In the parathyroid glands, the CaSR has been shown to couple to the G_q/11_ protein family (7), which activates phospholipase C (PLC), thereby increasing intracellular calcium (Ca^2+^_i_) and MAPK signaling responses (8, 9), which in turn leads to decreased parathyroid PTH secretion.

FHH is a genetically heterogeneous disorder with 3 recognized forms referred to as FHH types 1–3 (FHH1-3) (1). FHH1 (OMIM 145980) is caused by heterozygous loss-of-function mutations of the CaSR, which is encoded by the *CASR* gene on chromosome 3q21.1 (1). FHH2 (OMIM 145981) is due to heterozygous loss-of-function mutations of G-protein subunit α_11_ (Gα_11_), which is encoded by the *GNA11* gene on chromosome 19p13.3, and to date, 3 FHH2-associated mutations have been reported, comprising 2 missense mutations, Thr54Met and Leu135Gln, and an in-frame isoleucine deletion at codon 200 (Ile200del) (3, 10). FHH3 (OMIM 600740) is caused by heterozygous loss-of-function mutations of the adaptor protein-2 σ subunit (AP2σ), encoded by the *AP2S1* gene on chromosome 19q13.3, which is involved in the clathrin-mediated endocytosis of cell-surface proteins such as the CaSR (4, 11).

A mouse model for FHH1 has previously been generated by targeted germline disruption of the *Casr* gene, and heterozygous (*Casr^+/–^*) mice were shown to have a phenotype resembling that of FHH1 patients with elevated serum concentrations of calcium and PTH, and low urinary calcium excretion (12). In addition, homozygous (*Casr^–/–^*) mice had features of neonatal severe hyperparathyroidism (NSHPT), which is caused by biallelic inactivating CaSR mutations (1), and exhibited growth retardation and died within the first 30 days of life (12). An in vivo model is not available for FHH2, although mice with parathyroid-specific combined ablations of both the *Gna11* and *Gnaq* (encoding Gα_q_) genes have previously been reported to develop marked hypercalcemia and hyperparathyroidism (7). We therefore sought to establish a mouse model for FHH2 to define the in vivo role of Gα_11_ in Ca^2+^_o_ homeostasis and to undertake a more detailed characterization of the phenotype of this disorder, as limited information is available from the few FHH2 patients reported, to date (3, 10). In addition, a mouse model for FHH2 would facilitate evaluation of therapeutic drugs such as CaSR allosteric activators, also known as calcimimetics (13). To establish a mouse model for FHH2, due to a germline loss-of-function *GNA11* point mutation (3, 10), we screened a DNA archive of > 10,000 samples from male mice that had mutations induced by treating them with *N*-ethyl-*N*-nitrosourea (ENU), a chemical mutagen. ENU is an alkylating agent that introduces point mutations via transfer of an alkyl group from ENU to a DNA base, thus leading to mispairing and bp substitution during subsequent DNA replication (14, 15). ENU mutagenesis programs utilize two complementary approaches that are phenotype-driven and genotype-driven screens. In phenotype-driven screens, offspring of mutagenized mice are assessed for abnormalities in a hypothesis-generating strategy, which may elucidate new genes, pathways, and mechanisms for disease phenotypes (14, 15). Genotype-driven screens in which mutations in the gene of interest are sought are hypothesis driven and are feasible by available parallel archives of tissue-DNA and sperm samples from mutagenized male mice (14, 15). The archived tissue-DNA samples from the mutagenized male mice are used to search for the mutations in the gene of interest, and once these mutations are found, a sperm sample from the male mouse with the mutation is used for in vitro fertilization (IVF) of normal female mice to establish progeny with the mutation (14, 15). The probability of finding 3 or more variant alleles in an archive of tissue-DNA samples from > 5,000 ENU-mutagenized mice is > 90% (14). We sought for ENU-induced *Gna11* variants in tissue-DNA samples from > 10,000 male mice treated with ENU, with the aim of establishing a mouse model for FHH2.

## Results

### Identification and analysis of 5 Gna11 variants in ENU-mutagenized mice.

**Figure 1:**
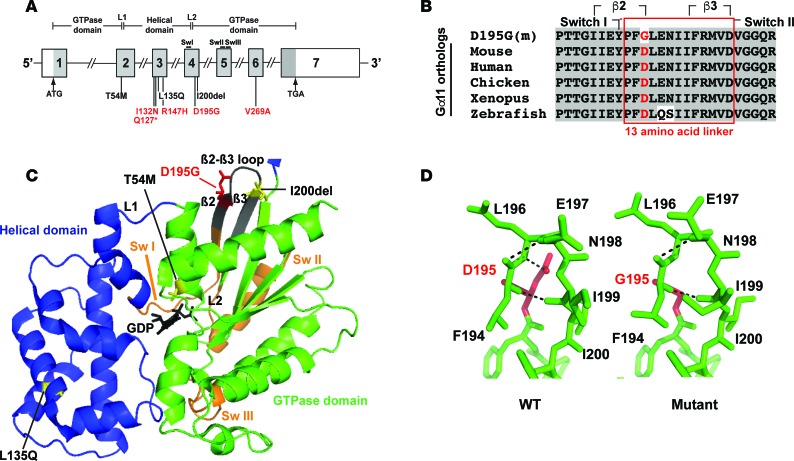
Structural characterization of the Asp195Gly Gα_11_ mutation. (**A**) Genomic organization of *Gna11* showing the location of reported human familial hypocalciuric hypercalcemia type 2 (FHH2) mutations (black) (3, 10) and *Gna11* variants identified in *N*-ethyl-*N*-nitrosourea– mutagenized (ENU-mutagenized) mice (red). The G-protein subunit α_11_ (Gα_11_) GTPase domain (encoded by exon 1, 5′ portion of exon 2, 3′portion of exon 4 and exons 5–7) is connected to the helical domain (encoded by the 3′portion of exon 2, exon 3, and 5′portion of exon 4) by the linker 1 (L1) and 2 (L2) peptides. The GTPase domain contains 3 flexible regions, termed switch regions I–III (SwI–SwIII). The Asp195Gly mutation is located within the GTPase domain and between the switch I and II regions. (**B**) Multiple protein sequence alignment of Gα_11_ residues comprising a 13–amino acid region that links the β2 strand of the switch I region with the β3 strand of the switch II region. Conserved residues are shown in gray. The WT (Asp, D) and mutant (m) (Gly, G) residues are shown in red. (**C**) Homology model of the GDP-bound Gα_11_ protein. The Gα helical (blue) and GTPase (green) domains and bound GDP nucleotide (black) are shown. Switch regions I–III are shown in orange. Previously reported residues mutated in FHH2 patients (3, 10) are shown in yellow. The mutated Asp195 residue (red) is located in a 13–amino acid region (gray) and adjacent to the β2-β3 loop. (**D**) Close-up view the β2-β3 hairpin loop region of WT and mutant Gα_11_ proteins showing the structural effects of the Asp195Gly mutant on hydrogen bonds (broken lines) within the hairpin loop. The Asp195Gly Gα_11_ mutant is predicted to result in a loss of a polar contact (hydrogen bond) between the Asp195 side chain and the backbone of the Glu197 (E197) residue. The one-letter amino acid codes indicate the following: D, aspartic acid; E, glutamic acid; F, phenylalanine; G, glycine; I, isoleucine; L, leucine; M, methionine; N, asparagine; Q, glutamine; and T, threonine.

An analysis using melting curve analysis (16) of tissue-DNA samples from > 10,000 ENU-mutagenized male mice of the 7 exons and 12 intron-exon boundaries of the *Gna11* gene revealed the presence of 5 *Gna11* variants, comprising c.379C>T, c.395T>A, c.440G>A, c.584A>G, and c.806T>C (numbering starts from ATG; Supplemental Table 1; supplemental material available online with this article; https://doi.org/10.1172/jci.insight.96540DS1; numbering in Supplemental Table 1 starts from 5′UTR.). These 5 *Gna11* variants predicted the occurrence of 4 missense variants (Ile132Asn, Arg147His, Asp195Gly, and Val269Ala) and 1 nonsense variant (Gln127Stop) (Figure 1A and Supplemental Figure 1). Bioinformatic analysis predicted all the Gα_11_ variants to be damaging and likely disease-causing (Supplemental Table 1). FHH2 has been reported to be caused by either an in-frame deletion or missense substitutions affecting Gα_11_ (3, 10), and we therefore further characterized only the 4 missense Gα_11_ variants identified in ENU-mutagenized mice. All of these 4 missense variants affected evolutionary-conserved residues (Figure 1B, Supplemental Table 1, and Supplemental Figure 1), and 2 variants (Asp195Gly and Val269Ala) were located in the Gα_11_ GTPase domain, which mediates GPCR binding, guanosine triphosphate (GTP) hydrolysis, and effector coupling. The other 2 variants (Ile132Asn and Arg147His) were located in the Gα_11_ helical domain, which stabilizes guanine nucleotide binding (Figure 1A and Supplemental Figure 1) (17). Three-dimensional (3-D) modeling using the reported crystal structure of the related Gα_q_ protein (18) predicted the Asp195Gly variant to disrupt polar contacts within the Gα_11_ GTPase domain (Figure 1, C and D), whereas the other missense variants were not predicted to alter intramolecular interactions within the Gα_11_ protein (Supplemental Figure 1). We therefore selected the Asp195Gly (D195G) variant for functional characterization for the following 4 reasons. First, this variant is located within the switch regions of the Gα_11_ GTPase domain (Figure 1, B and C), which are critical for mediating Gα-subunit conformational changes upon GTP binding and also for coupling to downstream effector proteins such as PLC (19, 20). Second, the Asp195Gly variant is situated within a 13 amino acid region (residues 193–205), which links switches I and II (Figure 1, B and C) and is the location of a reported FHH2-causing Gα_11_ mutation (Ile200del) (3). Third, this 13–amino acid linker region also contains the tetrapeptide β2-β3 loop (residues 196–199), which mediates G-protein–GPCR interactions (21) (Figure 1C), and our reported mutagenesis studies have shown that disruption of the Gα_11_ β2-β3 loop impairs signaling in CaSR-expressing cells (3). Fourth, 3-D modeling of the Asp195Gly Gα_11_ variant predicted that substitution of the WT Asp195 residue with the variant Gly195 residue would lead to a loss of a polar contact within the Gα_11_ β2-β3 loop, which would likely disrupt this tetrapeptide loop (Figure 1D) and thereby impair GPCR binding and Gα_11_ activation (3, 19, 20). These combined observations indicated that the Asp195Gly variant was highly likely to be a pathogenic mutation.

### In vitro functional characterization of the Asp195Gly Gα_11_ mutation.

**Figure 2:**
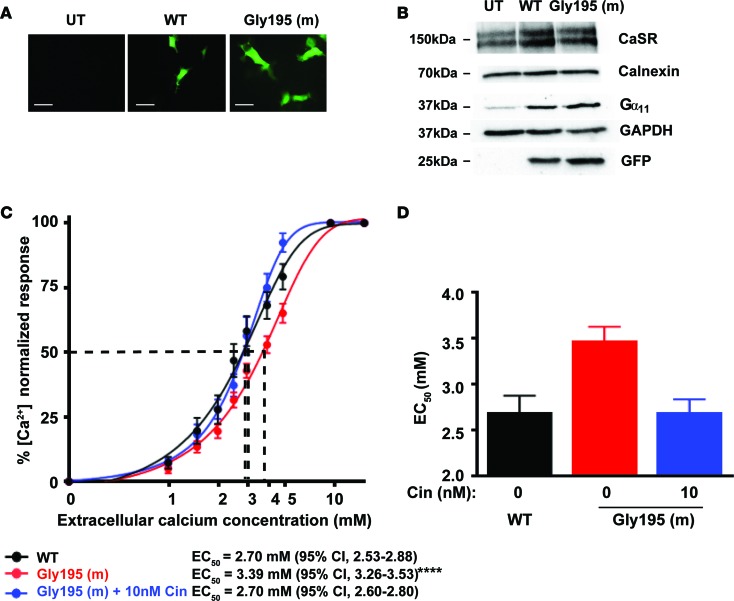
Ca^2+^_i_ responses of the Gly195 Gα_11_ mutant and in vitro effect of cinacalcet treatment. (**A**) Fluorescence microscopy of untransfected (UT) HEK293 cells stably expressing calcium-sensing receptor (CaSR) (HEK-CaSR) and of HEK-CaSR cells transiently transfected with WT (Asp195) or mutant (m) Gly195 pBI-CMV2-*GNA11*-GFP constructs. GFP expression in these cells indicates successful transfection and expression by these constructs. Scale bar: 10 μm. (**B**) Western blot analysis of lysates from HEK-CaSR cells used for intracellular calcium (Ca^2+^_i_) experiments. Transient transfection with WT or mutant Gly195 G-protein subunit α_11_ (Gα_11_) expression constructs resulted in overexpression of Gα_11_ and GFP, whereas UT cells showed only endogenous Gα_11_ protein expression. All cells expressed the CaSR. The calnexin and GAPDH proteins were used as loading controls. (**C**) Ca^2+^_i_ responses to changes in [Ca^2+^]_o_ of HEK-CaSR cells expressing WT or Gly195 Gα_11_ mutant proteins. The Ca^2+^_i_ responses are expressed as a percentage of the maximum normalized responses and shown as the mean ± SEM of 8 independent transfections. The Gly195 Gα_11_ mutant led to a rightward shift in the concentration-response curve (red line) compared with cells expressing WT Gα_11_ (black line). The addition of 10 nM cinacalcet (Cin) normalized the shift of the mutant concentration-response curve (blue line). (**D**) Histogram showing the mean half-maximal concentration (EC_50_) with 95% CI of WT cells (black), Gly195 mutant cells (red), and Gly195 mutant cells treated with 10 nM cinacalcet (blue). Statistical analysis was performed using the F-test. ****P < 0.0001 compared with WT.

To investigate the effects of these predicted Gα_11_ structural changes due to the Asp195Gly mutation on CaSR-mediated signaling, human embryonic kidney 293 (HEK293) cells stably expressing the CaSR (HEK-CaSR) were transiently transfected with pBI-CMV2-*GNA11-GFP* constructs expressing either the WT (Asp195) or variant (Gly195) Gα_11_ proteins, as reported (3). This bidirectional pBI-CMV2 vector allows for coexpression of Gα_11_ and GFP at equivalent levels (3). Expression of the CaSR, Gα_11_, and GFP was confirmed by fluorescence microscopy and/or Western blot analyses (Figure 2, A and B). The expression of Gα_11_ was shown to be similar in cells transiently transfected with WT or mutant proteins and to be greater than that observed in untransfected cells (Figure 2B). Moreover, the expression of mutant Gα_11_ in cells that endogenously express WT Gα_11_ (Figure 2B) corresponded to the heterozygous situation reported in FHH2 patients (3, 10). The Ca^2+^_i_ responses to alterations in [Ca^2+^]_o_ of cells expressing the different *GNA11* vectors were assessed using a multiwell assay that utilized the Fluo-4 Ca^2+^–binding dye, as reported (22). The Ca^2+^_i_ responses were shown to increase in a dose-dependent manner following stimulation with increasing [Ca^2+^]_o_ (Figure 2C). However, responses in mutant Gly195–expressing cells were significantly decreased compared with WT-expressing cells (Figure 2C). Thus, the mutant Gly195–expressing cells showed a rightward shift in the concentration-response curve (Figure 2C), with a significantly increased mean half-maximal response (EC_50_) of 3.39 mM (95% CI, 3.26–3.53 mM) compared with 2.70 mM (95% CI, 2.53–2.88 mM) for WT-expressing (Asp195) cells (*P* < 0.0001) (Figure 2, C and D). These results demonstrated that the Gα_11_ Asp195Gly mutation is a loss-of-function mutation, similar to mutations that lead to FHH2 (3, 10). We next investigated the ability of the CaSR allosteric activator, cinacalcet, to rectify this loss of function associated with the Asp195Gly Gα_11_ mutation. Cinacalcet was added to Gly195 mutant cells at a 10 nM concentration, as this dose has previously been reported to normalize the altered signaling responses associated with FHH2-causing Gα_11_ mutations in vitro (23). An assessment of Ca^2+^_i_ responses showed 10 nM cinacalcet to induce a leftward shift of the concentration-response curve of cells expressing the Gly195 mutant Gα_11_ protein (Figure 2C) and decrease their mean EC_50_ value to 2.70 mM (95% CI, 2.60–2.80 mM), a value that was indistinguishable from the EC_50_ of untreated WT cells (Figure 2, C and D). Thus, cinacalcet normalized the signaling responses of Gly195 mutant cells.

### In vivo functional analysis in mice harboring the germline Gna11 Asp195Gly mutation.

**Figure 3:**
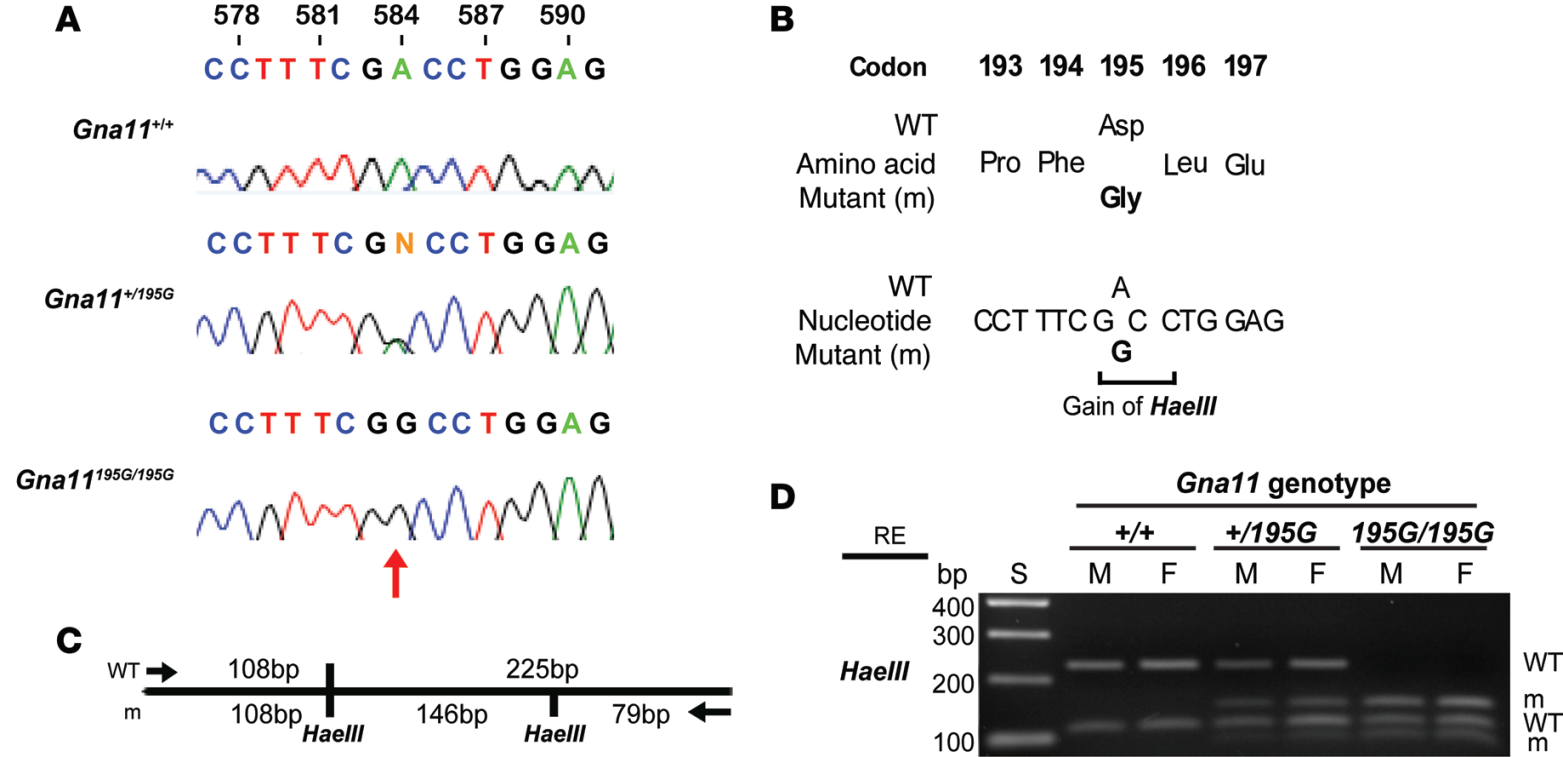
DNA sequence and restriction endonuclease analysis of the Asp195Gly Gα_11_ mutation. (**A**) DNA sequence analysis showing an A-to-G transition at c.584 (red arrow) within exon 3 of *Gna11* (numbering begins from ATG). The DNA sequence chromatograms show that WT (*Gna11^+/+^*) mice are homozygous A/A, the heterozygous mutant *Gna11^+/195G^* mice are A/G, and the homozygous mutant *Gna11^195G/195G^* mice are G/G. (**B**) This A-to-G transition was predicted to lead to a missense substitution of Asp, encoded by GAC, to Gly, encoded by GGC, at codon 195 and resulted in the gain of a *HaeIII* restriction endonuclease (RE) site (GG/CC). (**C**) Restriction maps showing that *HaeIII* digest would result in 2 products of 108 bp and 225 bp for the WT, and 3 products of 108 bp, 146 bp, and 79 bp for the mutant (m). (**D**) RE digest of *Gna11* exon 3 PCR products demonstrating that WT (*Gna11*+/+) mice are homozygous for the WT alleles, mutant *Gna11^+/195G^* mice are heterozygous and have WT and m alleles, and mutant *Gna11^195G/195G^* mice are homozygous for m alleles. M, male; F, female; S, size marker.

**Figure 4:**
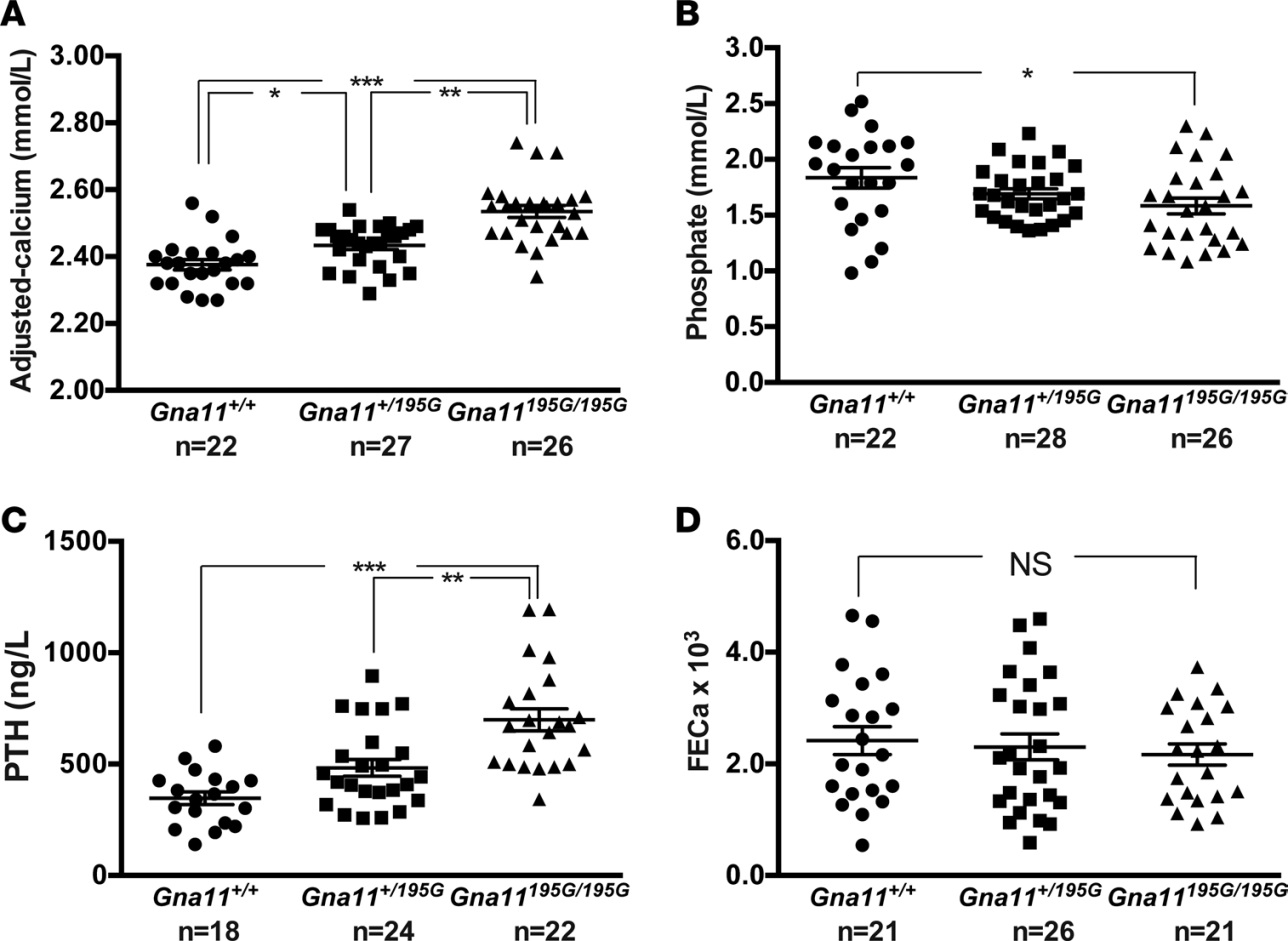
Calcitropic phenotype of *Gna11^+/+^*, *Gna11^+/195G^*, and *Gna11^195G/195G^* mice. (**A**) Plasma albumin–adjusted calcium concentrations, (**B**) plasma phosphate concentrations, (**C**) plasma parathyroid hormone (PTH) concentrations, and (**D**) fractional excretion of calcium (FECa) of *Gna11^+/+^* (circles), *Gna11^+/195G^* (squares), and *Gna11^195G/195G^* (triangles) mice, respectively. Combined data from males and females are shown. Mean ± SEM values for the respective groups are indicated by the solid bars. A Kruskal-Wallis test followed by Dunn’s test for nonparametric pairwise multiple comparisons were used for analysis of **A**–**D**. *P < 0.05, **P < 0.01, ***P < 0.001.

To investigate the in vivo effects of the Asp195Gly Gα_11_ mutation on Ca^2+^_o_ homeostasis, ENU mutagenesis–derived mice harboring this mutation were established on the C3H inbred genetic background (24). DNA sequence analysis confirmed the mutant mice to harbor a germline A-to-G transition at c.584A>G at codon 195 of the Gα_11_ protein resulting in an Asp (D) to Gly (G) missense substitution (Figure 3, A and B). This mutation led to a gain of a *HaeIII* restriction endonuclease site (Figure 3C), which was used to confirm the presence of the mutation (Figure 3D) and to genotype the subsequent generations of mice. Heterozygous-affected (*Gna11^+/195G^*) mice were healthy and fertile, and an analysis of offspring bred from crosses of *Gna11^+/195G^* mice yielded homozygous-affected (*Gna11^195G/195G^*) mice and significant deviations from the Mendelian inheritance expected ratio of 1:2:1 for the WT (*Gna11^+/+^*), *Gna11^+/195G^*, and *Gna11^195G/195G^* genotypes were not observed among the weaned mice, thereby indicating that the homozygous *Gna11^195G/195G^* mice were viable and did not have embryonic or neonatal lethality (Table 1). Moreover, *Gna11^195G/195G^* mice had a normal body weight compared with WT (*Gna11^+/+^*) and *Gna11^+/195G^* littermates (Table 2). Thus, *Gna11^195G/195G^* mice did not have evidence of growth retardation or neonatal lethality to suggest an NSHPT phenotype. However, plasma biochemical analysis revealed *Gna11^+/195G^* and *Gna11^195G/195G^* mice to be significantly hypercalcemic compared with *Gna11^+/+^* mice (Figure 4A). Moreover, *Gna11^195G/195G^* mice had significantly reduced plasma phosphate concentrations and raised plasma PTH concentrations when compared with *Gna11^+/+^* mice, whereas *Gna11^+/195G^* mice had plasma phosphate and PTH concentrations that were similar to those of *Gna11^+/+^* mice (Figure 4, B and C). Furthermore, the fractional excretion of calcium was not altered in *Gna11^+/195G^* or *Gna11^195G/195G^* mice compared with *Gna11^+/+^* mice (Figure 4D and Table 3). However, there were sex differences in these calcitropic phenotypes, as follows. Female *Gna11^195G/195G^* mice were significantly more hypercalcemic than male *Gna11^195G/195G^* mice and female *Gna11^+/195G^* mice (Table 2 and Supplemental Figure 2). In addition, female *Gna11^195G/195G^* mice, but not the *Gna11* mutant males, had significant hypophosphatemia, with a significant reduction in the tubular maximum reabsorption of phosphate (Table 3) and a raised alkaline phosphatase activity compared with female *Gna11^+/+^* mice (Table 2 and Supplemental Figure 2). Significant differences were not observed in plasma electrolytes, urea and creatinine concentrations, or 1,25-dihydroxyvitamin D or fibroblast growth factor-23 (FGF-23) concentrations in male or female *Gna11^+/195G^* and *Gna11^195G/195G^* mice, when compared with respective *Gna11^+/+^* mice (Table 2). The fractional excretions of sodium and potassium were also not different between male and female mutant mice and respective *Gna11^+/+^* mice (Table 3). Finally, whole body dual-energy X-ray absorptiometry (DXA) did not reveal significant differences in the bone mineral content or bone mineral density (BMD) between male and female mutant mice and respective *Gna11^+/+^* mice (Table 4).

**Figure 5:**
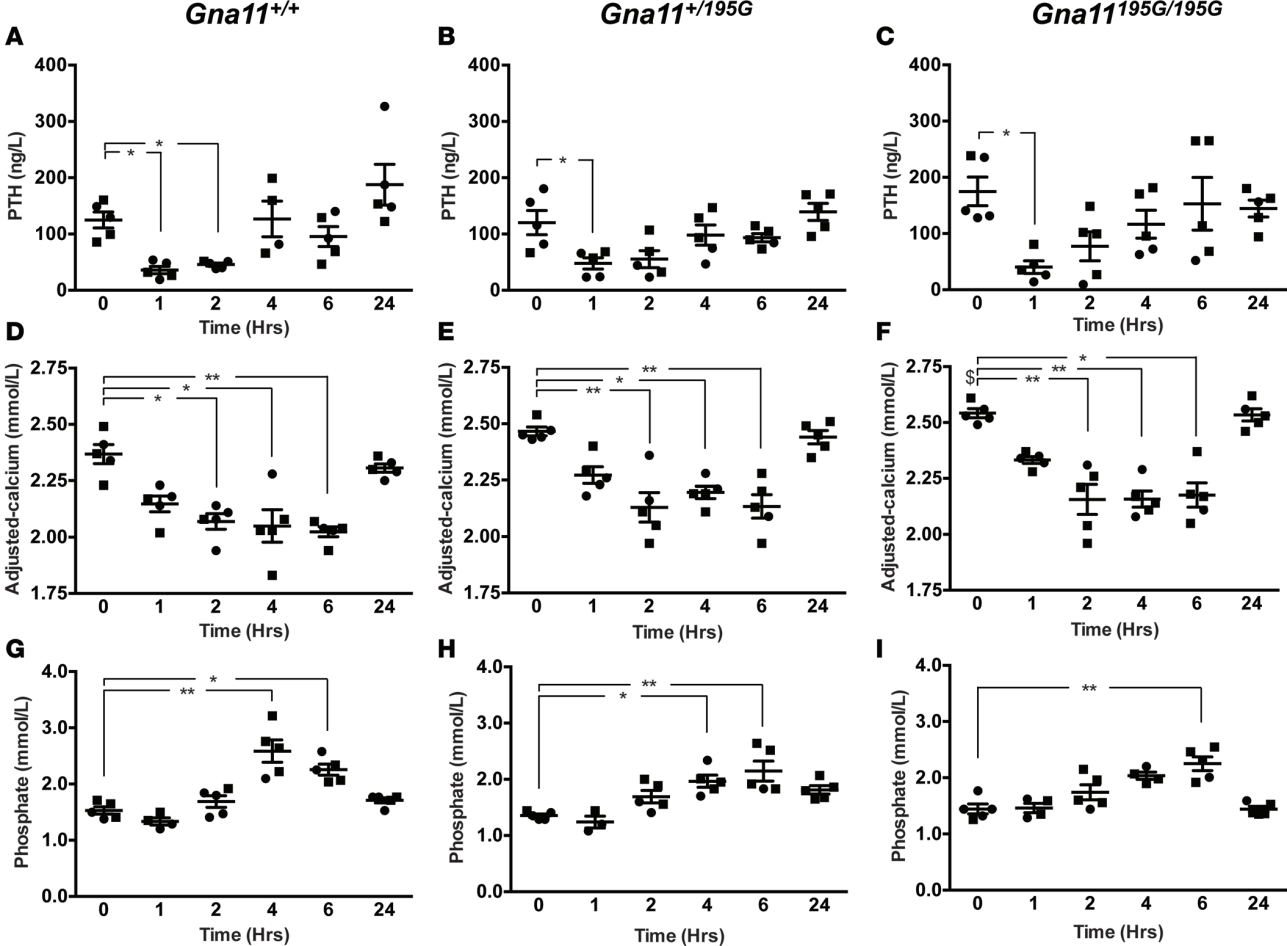
In vivo effect of cinacalcet on plasma PTH, calcium, and phosphate concentrations of *Gna11^+/+^*, *Gna11^+/195G^*, and *Gna11^195G/195G^* mice. (**A–C**) Plasma parathyroid hormone (PTH), (**D–F**) plasma albumin–adjusted calcium, and (**G–I**) plasma phosphate concentrations are shown at 0, 1, 2, 4, 6, and 24 hours following oral gavage administration of a single 30 mg/kg cinacalcet dose. Mean values for the respective groups are indicated by solid bars. *n* = 4–5 mice per study time point. Squares, males; circles, females. A Kruskal-Wallis test followed by Dunn’s test for nonparametric pairwise multiple comparisons were used for analysis of **A**–**I**. *P < 0.05, **P < 0.01 compared with respective untreated mice. ^$^Untreated *Gna11^195G/195G^* mice were significantly (*P* < 0.05) hypercalcemic compared with untreated *Gna11^+/+^* mice.

To determine whether the hypercalcemia of *Gna11^+/195G^* and *Gna11^195G/195G^* mice may be improved by in vivo calcimimetic treatment, we administered cinacalcet to WT and mutant mice. A pilot dose-ranging study in WT mice showed that a single oral gavage 30 mg/kg dose of cinacalcet significantly lowered plasma PTH concentrations, when compared with vehicle-treated mice (Supplemental Figure 3). This dose (30mg/kg) of cinacalcet was therefore administered by oral gavage to *Gna11^+/+^*, *Gna11^+/195G^*, and *Gna11^195G/195G^* mice, and plasma samples were then taken at 0, 1, 2, 4, 6, and 24 hours after dose for the measurement of PTH, calcium, phosphate, urea, creatinine, and albumin concentrations. Administration of cinacalcet significantly decreased plasma PTH concentrations in *Gna11^+/+^*, *Gna11^+/195G^*, and *Gna11^195G/195G^* mice by ≥ 60% at 1 hour after dose, with values returning to baseline by 4–6 hours after dose (Figure 5, A–C), and it significantly reduced plasma albumin–adjusted calcium concentrations in *Gna11^+/+^*, *Gna11^+/195G^*, and *Gna11^195G/195G^* mice between 2–6 hours after dose, with values returning to baseline by 24 hours after dose (Figure 5, D–F). Cinacalcet treatment also resulted in a transient rise in plasma phosphate concentrations in *Gna11^+/+^*, *Gna11^+/195G^*, and *Gna11^195G/195G^* mice (Figure 5, G–I) but was not associated with any increases in plasma concentrations of urea or creatinine (Supplemental Figure 4). Thus, these studies demonstrated that cinacalcet is effective in vivo and can reduce raised plasma calcium and PTH concentrations observed in *Gna11* mutant mice with a loss-of-function Gα_11_ mutation, which is representative of FHH2.

## Discussion

We have established a mouse model for FHH2, and this will enable the calcitropic roles of Gα_11_ to be further evaluated and also facilitate further pathophysiological studies that are difficult to pursue in the few reported patients with this condition. Our results revealed that heterozygous-affected (*Gna11^+/195G^*) mice had a similar plasma biochemical phenotype to that reported for FHH2 patients, who also harbor heterozygous loss-of-function Gα_11_ mutations (Table 5) (3, 10). Thus, *Gna11^+/195G^* mice had mild hypercalcemia in association with normal plasma PTH concentrations; they also had no alterations in the plasma concentrations of phosphate and creatinine, or in alkaline phosphatase activity, which is consistent with the reported phenotype of FHH2 patients (Table 5) (3, 10). *Gna11^+/195G^* mice additionally had normal plasma magnesium concentrations, which is consistent with one reported FHH2 proband (3) but which contrasts with the hypermagnesemia reported in a multigenerational FHH2 kindred (3). A key finding of this study is that *Gna11^+/195G^* and *Gna11^195G/195G^* mice had no alterations in urinary calcium excretion, and this would be consistent with studies of FHH2 patients, which have reported that not all FHH2 patients have a low fractional excretion of calcium (Table 5) (3, 10). The absence of a urinary calcium phenotype in *Gna11^+/195G^* and *Gna11^195G/195G^* mice is also consistent with the reported findings in mice and humans harboring germline gain-of-function Gα_11_ mutations that is associated with hypocalcemia and reduced plasma PTH concentrations but with mild or no alterations in urinary calcium excretion (25–27). These studies highlight a potential difference in the calcitropic phenotype of disorders caused by germline Gα_11_ mutations and that of disorders caused by germline CaSR mutations, and they suggest that the Gα_11_ protein may not play a major role in the renal handling of calcium. Thus, it remains to be established whether hypocalciuria represents a major component of the FHH2 disorder in humans. Furthermore, DXA analysis did not reveal any alterations in the BMD values of *Gna11^195G/195G^* mice, which also suggests that the Gα_11_ protein may not influence bone mass.

Our studies of homozygous-affected (*Gna11^195G/195G^*) mice have highlighted the importance of Gα_11_ for parathyroid gland function and PTH secretion, as *Gna11^195G/195G^* mice had more pronounced hypercalcemia and hypophosphatemia, and significantly raised plasma PTH concentrations, consistent with primary hyperparathyroidism (28). Moreover, female *Gna11^195G/195G^* mice also had significant elevations of plasma alkaline phosphatase activity, which is consistent with an elevated bone turnover associated with this likely primary hyperparathyroidism. However, the hypercalcemic phenotype of *Gna11^195G/195G^* mice was, in general, milder than that observed in humans or mice harboring biallelic loss-of-function CaSR mutations, which typically lead to the life-threatening disorder of NSHPT (12, 24). A possible explanation for the milder hypercalcemic phenotype observed in the *Gna11^195G/195G^* mice is that the loss of Gα_11_ function caused by the Asp195Gly mutation in vivo was partially compensated by the WT Gα_q_ protein, which in the parathyroid glands continues to mediate signal transduction by the CaSR. Indeed, the importance of the Gα_11_ and Gα_q_ proteins for parathyroid gland function has been demonstrated by studies of mice with a parathyroid-specific ablation of both Gα_11_ and Gα_q_, which have been reported to develop features of NSHPT such as severe hypercalcemia, skeletal demineralization, growth retardation, and early postnatal death (7). The hypercalcemia observed in *Gna11^195G/195G^* mice was more severe in females compared with males, and such sex differences have not previously been reported in studies of FHH patients. However, sex differences have been noted in primary hyperparathyroidism patients, with females being more commonly affected than males (29). Moreover, estrogen may play a role in the pathogenesis and severity of primary hyperparathyroidism, as highlighted by a study that showed the potential involvement of estrogen signaling in parathyroid function and disease (30); such effects may have contributed to the more severe hypercalcemia of female *Gna11^195G/195G^* mice.

There is currently no effective treatment for FHH2, and we therefore evaluated the therapeutic potential of cinacalcet, which is a licensed CaSR-positive allosteric modulator (13), for this condition. In vitro studies have previously reported that nanomolar concentrations of cinacalcet can successfully rectify the altered signaling responses of HEK-CaSR cells expressing FHH2-associated Gα_11_ mutant proteins (23). Consistent with these findings, our study showed a 10 nM concentration of cinacalcet to normalize the Ca^2+^_i_ responses of HEK-CaSR cells expressing the mutant Gly195 Gα_11_ protein. Moreover, oral administration of a single 30 mg/kg cinacalcet dose led to a transient suppression of PTH secretion in *Gna11^+/195G^* and *Gna11^195G/195G^* mice, and this was associated with a sustained reduction in plasma calcium concentrations, which lasted for ≥ 6 hours. This dose of cinacalcet was well tolerated in the mice and did not lead to hypocalcemia, with mean plasma calcium concentrations remaining at > 2.0 mmol/l. However, transient hyperphosphatemia was noted in cinacalcet-treated mice, which was likely to be a consequence of suppressed PTH secretion (31). These results suggest that calcimimetics such as cinacalcet will likely be of benefit for FHH2 patients, who also harbor loss-of-function Gα_11_ mutations (23).

In summary, we have established a mouse model for FHH2 and have shown the in vivo efficacy of cinacalcet in reducing plasma calcium and PTH concentrations, thereby illustrating the potential utility of this CaSR allosteric modulator for the treatment of hypercalcemia in patients with FHH2.

## Methods

### Animals.

ENU-treated G0 C57BL/6J male mice (The Jackson Laboratory) were mated to C3H/HeH (C3H) mice (MRC Harwell) to produce G1 progeny, and tissue-DNA samples from > 10,000 G1 ENU mutagenized male mice — together with their sperm — was archived, as reported (14). These tissue-DNA samples were used to identify *Gna11* variants by melt curve analysis of PCR products utilizing a Lightscanner and gene-specific primers (BioFire Diagnostics Inc.), and sperm from mice with *Gna11* variants was used for IVF to generate G2 progeny on a C3H background strain, as reported (16, 24). Heterozygous-affected (*Gna11^+/195G^*) mutant male and female mice were intercrossed to generate homozygous (*Gna11^195G/195G^*) mice, which were studied along with their *Gna11^+/195G^* and WT (*Gna11^+/+^*) littermates. All study mice were housed in a controlled environment at the MRC Harwell Institute in accordance with UK Home Office and MRC Welfare guidance. Mice were fed on a standard diet (Rat and Mouse number 3, Special Diet Services) that contained 1.15% calcium, 0.58% phosphate, and 4089 IU/kg of vitamin D, and they were provided with water ad libitum (25, 32).

### Compounds.

Cinacalcet (AMG-073 HCL) was obtained from Cambridge Bioscience (catalog CAY16042) and dissolved in a 20% aqueous solution of 2-hydroxypropyl-β-cyclodextrin (MilliporeSigma, catalog H107) prior to use in in vitro and in vivo studies.

### DNA sequence analysis.

Genomic DNA was isolated from auricular biopsies using DNA extraction buffer (10 mM NaCl, 20 mM Tris-HCl, pH 8.0, 1 mM EDTA, 10% SDS; MilliporeSigma) and Proteinase K solution (Thermo Fisher Scientific) (25). Genomic DNA was used with *Gna11* gene–specific primers (MilliporeSigma) to perform PCR amplification, followed by dideoxynucleotide sequencing using the BigDye Terminator v3.1 Cycle Sequencing Kit and an automated detection system (ABI 3730 Automated capillary sequencer, Thermo Fisher Scientific), as reported (25). MutationTasting (http://www.mutationtaster.org/) and Polyphen-2 software was used to predict variant pathogenicity (33, 34). The *Gna11* germline mutation was confirmed by *HaeIII* restriction endonuclease analysis (New England Biolabs), as previously described (3, 4).

### Protein sequence alignment and 3-D modeling.

Protein sequences of Gα_11_ orthologs and paralogs were aligned with Clustal Omega (35). The PyMOL Molecular Graphics System (Version 1.2r3pre, Schrödinger LLC) was used for structural modeling based on the complexed crystal structure of Gα_q_, which has 90% identity with Gα_11_ at the amino acid level (Protein Data Bank, accession no. 4GNK; https://www.rcsb.org/pdb/home/home.do) (18). The effect of the Gα_11_ mutations upon Gα_11_ structure was modeled using the PyMod plug-in and Modeller (36).

### Cell culture and transfection.

Functional studies were undertaken using a human *GNA11* construct (3), as the human and mouse Gα_11_ proteins share an overall amino acid identity of 98% (25) and are 100% identical in the region surrounding the mutated site. The Gly195 mutation was introduced by site-directed mutagenesis (QuikChange Lightning, Agilent Technologies) into a pBI-CMV2-*GNA11-GFP* expression construct, as reported (3), and WT and mutant pBI-CMV2-*GNA11-GFP* constructs were transiently transfected into HEK293 cells stably expressing the full-length human *CASR* cDNA (HEK-CaSR), as described (3). HEK-CaSR cells were maintained in DMEM-Glutamax media (Thermo Fisher Scientific) with 10% FBS (Gibco) and 400 μg/ml geneticin (Thermo Fisher Scientific) at 37°C, 5% CO_2_ (3). Successful transfection was confirmed by visualizing GFP fluorescence using an Eclipse E400 fluorescence microscope with an epifluorescence filter, and images were captured using a DXM1200C digital camera and NIS Elements software (Nikon) (3, 11). The expression of Gα_11_, CaSR, GFP, calnexin, and GAPDH proteins was confirmed by Western blot analyses using anti-Gα_11_ (D-6, sc-390382, Santa Cruz Biotechnologies Inc.), anti-CaSR (5C10, ADD; ab19347; Abcam), anti-GFP (B-2, sc-9996, Santa Cruz Biotechnologies Inc.), anti-calnexin (AB2301, Millipore), and anti-GAPDH (AM4300, Ambion) antibodies, respectively. The Western blots were visualized using an Immuno-Star Western C kit (Bio-Rad) on a Bio-Rad Chemidoc XRS+ system (3, 10).

### Measurement of Ca^2+^_i_ responses.

The Ca^2+^_i_ responses of HEK-CaSR cells expressing WT or mutant Gα_11_ proteins were measured by Fluo-4 calcium assays adapted from methods previously published (22). HEK-CaSR cells were plated in poly-L-lysine–treated black-walled 96-well plates (Corning) and were transiently transfected with 1,000 μg/ml pBI-CMV2-*GNA11-GFP*. On the following day, cells were incubated in serum-free media for 2 hours and then loaded with the Fluo-4 Ca^2+^–binding dye, prepared according to manufacturer’s instructions (Invitrogen). Cells were loaded for 40 minutes at 37°C. Then, either a 20% aqueous solution of 2-hydoxypropyl-β-cyclodextrin (vehicle) or 10 nM cinacalcet was added, and cells were incubated for a further 20 minutes at 37°C (19). Baseline measurements were made, and increasing doses of CaCl_2_ were injected into each well, using the PHERAstar microplate reader (BMG Labtech) automated system. Changes in Ca^2+^_i_ were recorded on a PHERAstar instrument (BMG Labtech) at 37°C with an excitation filter of 485 nm and an emission filter of 520 nm. The peak mean fluorescence ratio of the transient response after each individual stimulus was measured using MARS data analysis software (BMG Labtech) and expressed as a normalized response. Nonlinear regression of concentration-response curves was performed with GraphPad Prism using the normalized response at each [Ca^2+^]_o_ for each separate experiment for the determination of the EC_50_ values.

### Metabolic cage studies and biochemical analysis.

Thirteen- to 15-week-old mice were individually housed in metabolic cages (Techniplast) for 24 hours with free access to food and water. Mice were allowed to acclimatize to their environment over a 72-hour period, as described (37), prior to collection of 24-hour urine samples. Twenty-four–hour urine samples were collected in tubes containing sodium azide, and blood samples were collected from the lateral tail vein under topical local anesthesia (38) or from the retro-orbital vein into lithium heparin Microvette tubes (Sarstedt) following terminal isoflurane anesthesia, as described (25, 32). Plasma and urine were analyzed for sodium, potassium, total calcium, phosphate, magnesium, urea, creatinine, and alkaline phosphatase activity on a Beckman Coulter AU680 analyzer (25, 32). Plasma calcium was adjusted for variations in albumin concentrations using the formula: plasma calcium (mmol/l) – ([plasma albumin – mean albumin (g/l) of respective male and female WT mice] × 0.02), as reported (39). Hormones were measured as follows: PTH using a 2-site ELISA specific for mouse intact PTH (Immutopics); 1,25-dihydroxyvitamin D by a 2-step process involving purification by immunoextraction and quantification by enzyme immunoassay (Immunodiagnostic Systems); and FGF-23 using a 2-site ELISA kit (Kainos Laboratories), as described (25, 39). The fractional excretion of sodium, potassium, and calcium were calculated using the formula U_x_/P_x_ × P_Cr_/U_Cr_, where U_x_ is the urinary concentration of the filtered substance (substance *x*) in mmol/l, P_x_ is the plasma concentration of substance *x* in mmol/l, U_Cr_ is the urinary concentration of creatinine in mmol/l, and P_Cr_ is the plasma concentration of creatinine in mmol/l. The ratio of tubular maximum reabsorption of phosphate to GFR (TmP/GFR) was calculated using the following formula: P_Pi_ × (1 – [U_Pi_/P_Pi_ × P_Cr_/U_Cr_ ]), where P_Pi_ is the plasma concentration of phosphate and U_Pi_ is the urine concentration of phosphate (25, 32).

### Skeletal imaging.

Bone mineral content and density were assessed by whole body DXA scanning, which was performed on mice anesthetized by inhaled isoflurane and using a Lunar Piximus densitometer (GE Medical Systems), as reported (25). DXA images were analyzed using Piximus software, as reported (25).

### Statistics.

All in vitro studies involved 8 biological replicates. Statistical comparisons of the Ca^2+^_i_ EC_50_ responses were undertaken using the *F*-test, as reported (3). For the in vivo studies, a Kruskal-Wallis test was undertaken for multiple comparisons, and any significant differences identified were further assessed using the Dunn’s test for nonparametric pairwise multiple comparisons (25). All analyses were performed using GraphPad Prism (GraphPad), and a value of *P* < 0.05 was considered significant for all analyses.

### Study approval.

Animal studies were approved by the MRC Harwell Institute Ethical Review Committee and were licensed under the Animal (Scientific Procedures) Act 1986, issued by the UK Government Home Office Department (PPL30/2433 and PPL30/3271).

## Author Contributions

SAH, FMH, CMG, MAN, SDMB, RDC, and RVT designed research studies; SAH, CMG, AP, MS, TAH, and SW conducted experiments; SAH, FMH, CMG, and SEP acquired and analyzed data; and SAH, FMH, CMG, and RVT wrote the manuscript.

## Supplementary Material

Supplemental data

## Figures and Tables

**Table 5:**
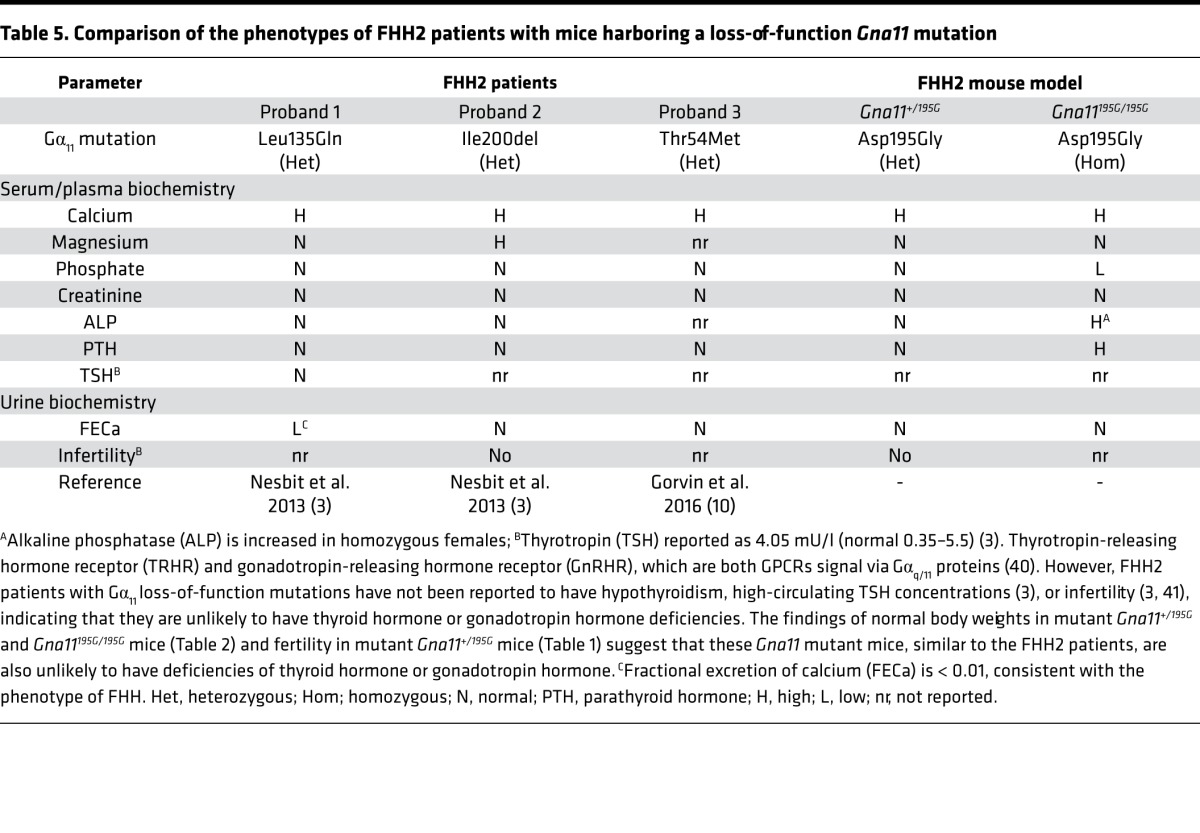
Comparison of the phenotypes of FHH2 patients with mice harboring a loss-of-function *Gna11* mutation

**Table 4:**
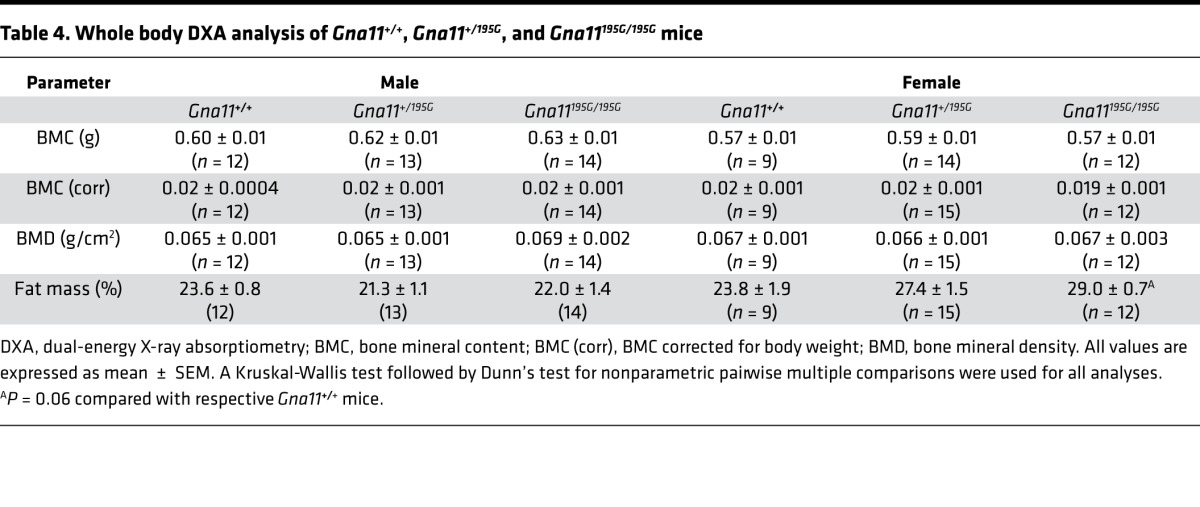
Whole body DXA analysis of *Gna11^+/+^*, *Gna11^+/195G^*, and *Gna11^195G/195G^* mice

**Table 3:**
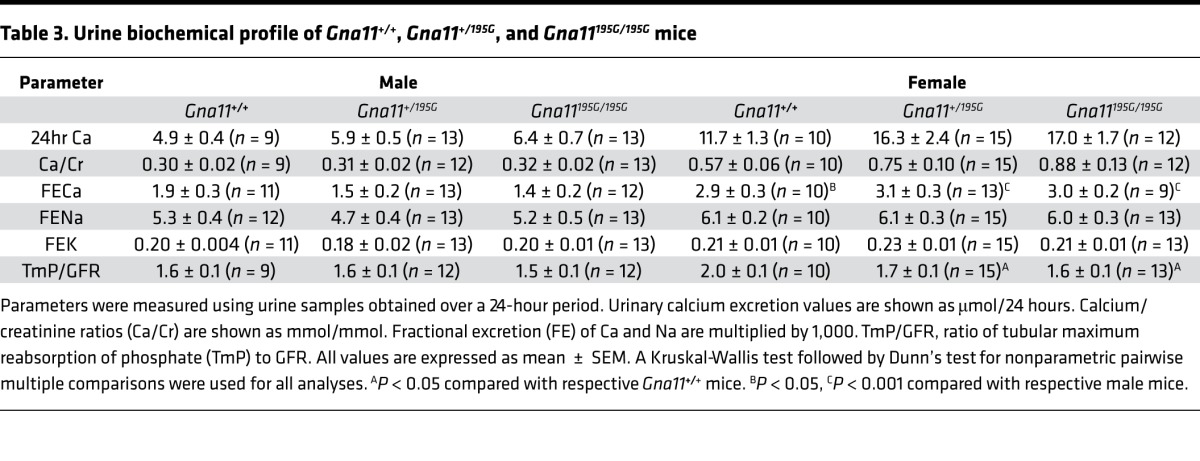
Urine biochemical profile of *Gna11^+/+^*, *Gna11^+/195G^*, and *Gna11^195G/195G^* mice

**Table 2:**
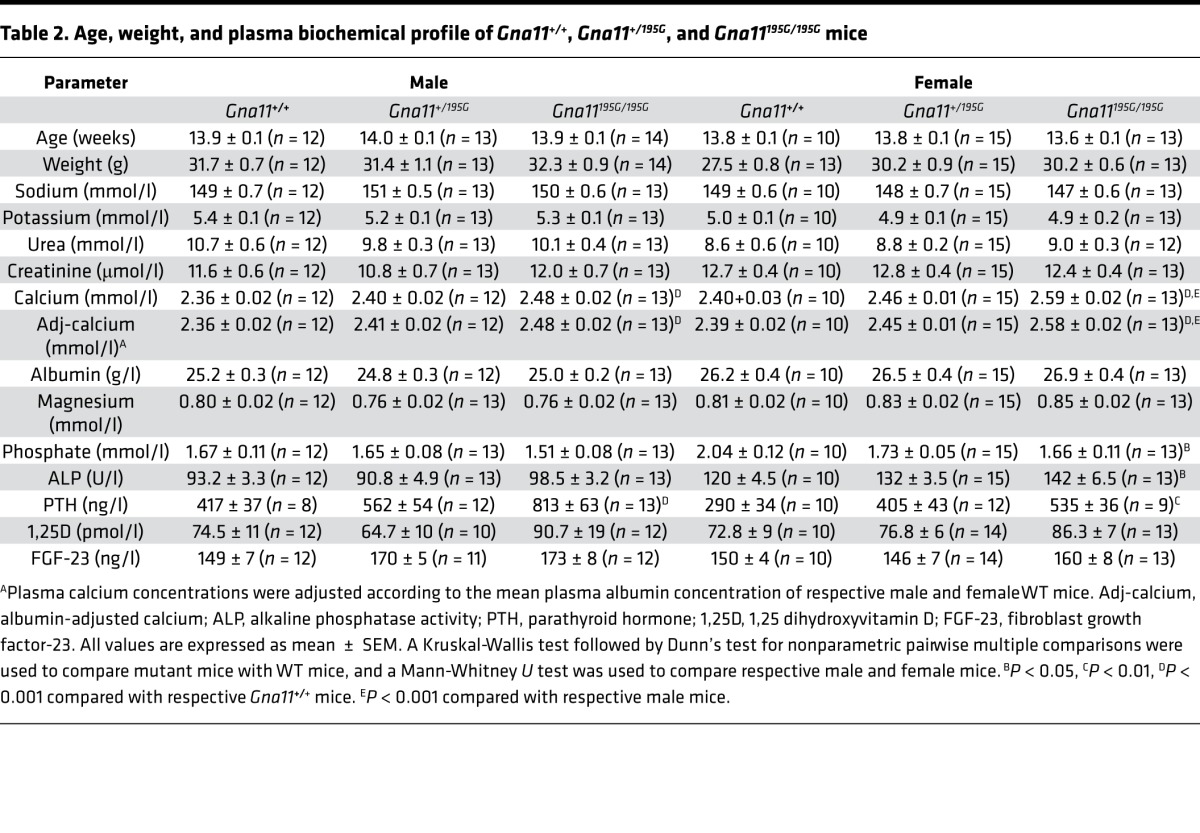
Age, weight, and plasma biochemical profile of *Gna11^+/+^*, *Gna11^+/195G^*, and *Gna11^195G/195G^* mice

**Table 1:**
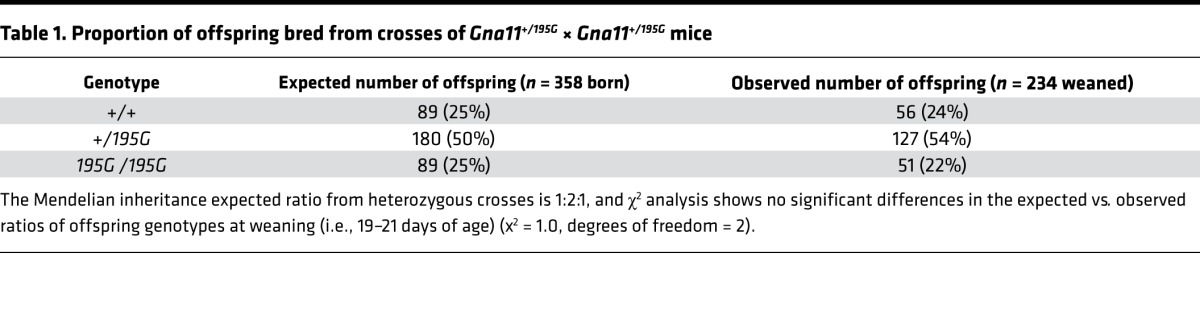
Proportion of offspring bred from crosses of *Gna11^+/195G^* × *Gna11^+/195G^* mice

## Version 1 10/19/2017

Electronic publication
