# Supplementary material for: Cinacalcet corrects hypercalcemia in mice with an inactivating Gα11 mutation
Source: JCI Insight. 2017 Oct 19;2(20):e96540. doi: 10.1172/jci.insight.96540 (PMC5846897; doi:10.1172/jci.insight.96540)
Supplement: Supplemental data [file jciinsight-2-96540-s001.pdf]

Supplementary Table 1. *Gna11* missense variants identified in ENU mutagenized mice.

ENU mutation		Frequency in population (%)[a]	Conservation[b]	Polyphen-2 prediction[c]	Location in $G\alpha_{11}$[d]
**Nucleotide**	**Predicted Change**				
c.621C>T	Gln127Stop	0	2/4	0.9999 (Disease-causing)	Helical domain (αC-helix)
c.637T>A	Ile132Asn	0	4/4	0.9999 (Disease-causing)	Helical domain (αC-helix)
c.682G>A	Arg147His	0	4/4	0.9999 (Disease-causing)	Helical domain (αD-αE loop)
c.826A>G	Asp195Gly	0	4/4	0.9999 (Disease-causing)	GTPase domain (β2-strand)
c.1048T>C	Val269Ala	0	4/4	0.9999 (Disease-causing)	GTPase domain (β5-strand)

[a]Frequency of variant in population derived from ExAc database (http://exac.broadinstitute.org/) (accessed June 2017), representing exomes from 60,706 unrelated individuals. [b]Conservation of WT residue in $G\alpha_{11}$ orthologs from four species (human, chicken, xenopus, zebrafish). [c]Polyphen-2 scores predict the effect of amino acid substitutions based on sequence homology, protein databank structures, and pfam annotations (31). It gives a qualitative score of probably damaging, possibly damaging, benign, or unknown and quantitative scores are based on the probability that the change is damaging, i.e. the nearer to 0 the more benign. A mutation is classified as probably damaging if the score is >0.85, and possibly damaging if the score is >0.15 (32). [d]Location in $G\alpha_{11}$ based on structural modeling using the published crystal structures of the highly related $G\alpha_q$ protein (Protein Data Bank, accession number 4GNK (18).

Supplementary Figures

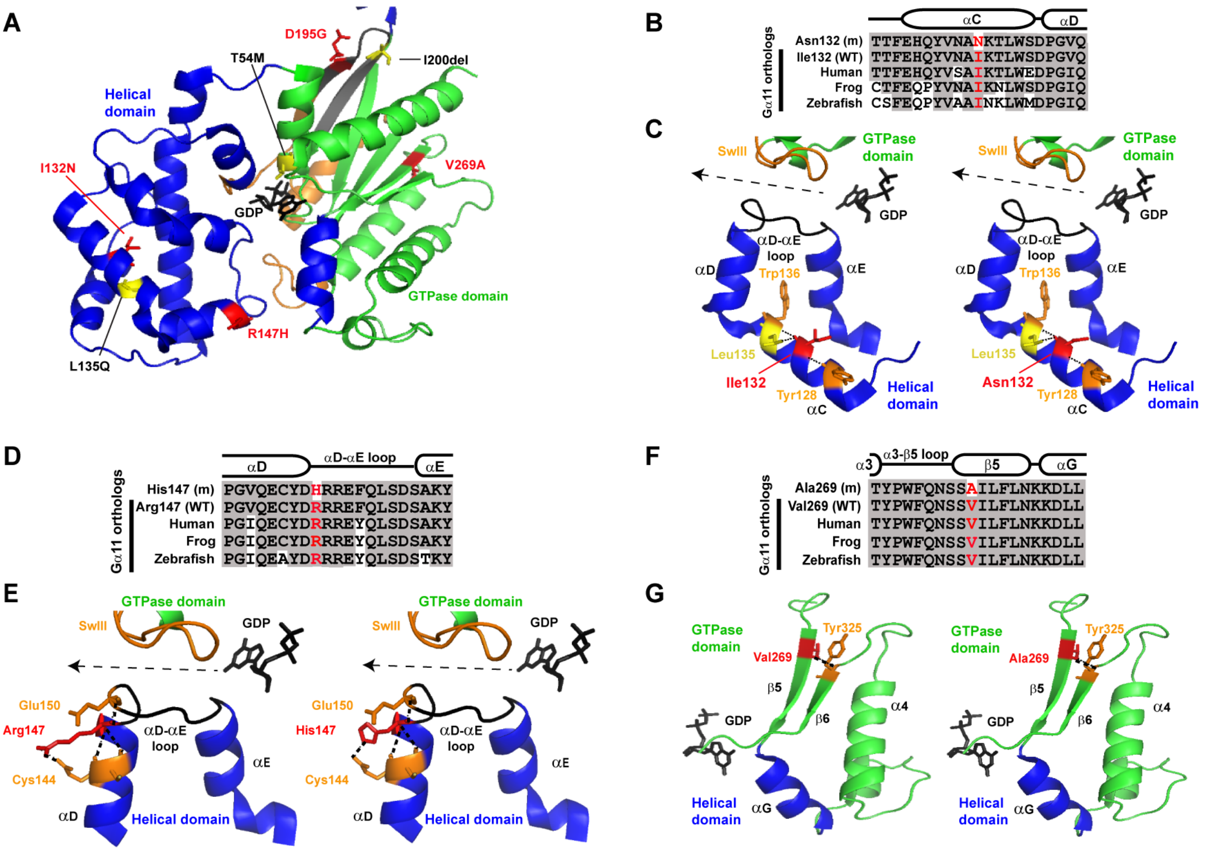

Supplementary Figure 1. Structural characterization of the Gα_{11} variants identified in ENU mutagenized mice. (**A**) Homology model of the GDP-bound Gα_{11} protein. The Gα helical (blue) and GTPase (green) domains, and bound GDP nucleotide (black) are shown. Switch regions I-III are shown in orange. Previously reported residues mutated in FHH2 (3, 10) patients are shown in yellow and the variants identified in ENU mutagenized mice are shown in red. (**B**) Multiple protein sequence alignment of Gα_{11} residues comprising the αC and αD helices of the Gα_{11} helical domain. Conserved residues are shown in gray. The WT (Ile, I) and mutant (m) (Asn, N) residues are shown in red. (**C**) Close-up view of the αC-αE helices, which are located close to the SwIII region which facilitates GDP-GTP exchange (indicated by the black arrow). The Ile132 residue within αC forms hydrogen bonds (broken lines) with three adjacent residues (shown in orange and yellow); however, the ENU Asn132 variant does not affect these bonds. (**D**) Multiple protein sequence alignment of Gα_{11} residues comprising the αD-αE helices of the Gα_{11} helical domain. The WT (Arg, R) and mutant (m) (His, H) residues are shown in red. (**E**) Close-up view of the αD-αE loop, located close to the SwIII region. The Arg147 Gα_{11} residue forms polar contacts with adjacent Cys144 and Glu150 residues (orange); however, the ENU His147 variant has no effect on these bonds. (**F**) Multiple protein sequence alignment of Gα_{11} residues comprising the β5 strand and α3 and αG helices of Gα_{11}. The WT (Val, V) and mutant (m) (Ala, A) residues are shown in red. (**G**) Close-up view of the α4 helix and β5-β6 strands of the Gα_{11} GTPase domain. The Val269 Gα_{11} residue, located in the β5 strand forms a hydrogen bond with the Tyr325 residue on β6, which is not affected by the ENU Ala269 variant.

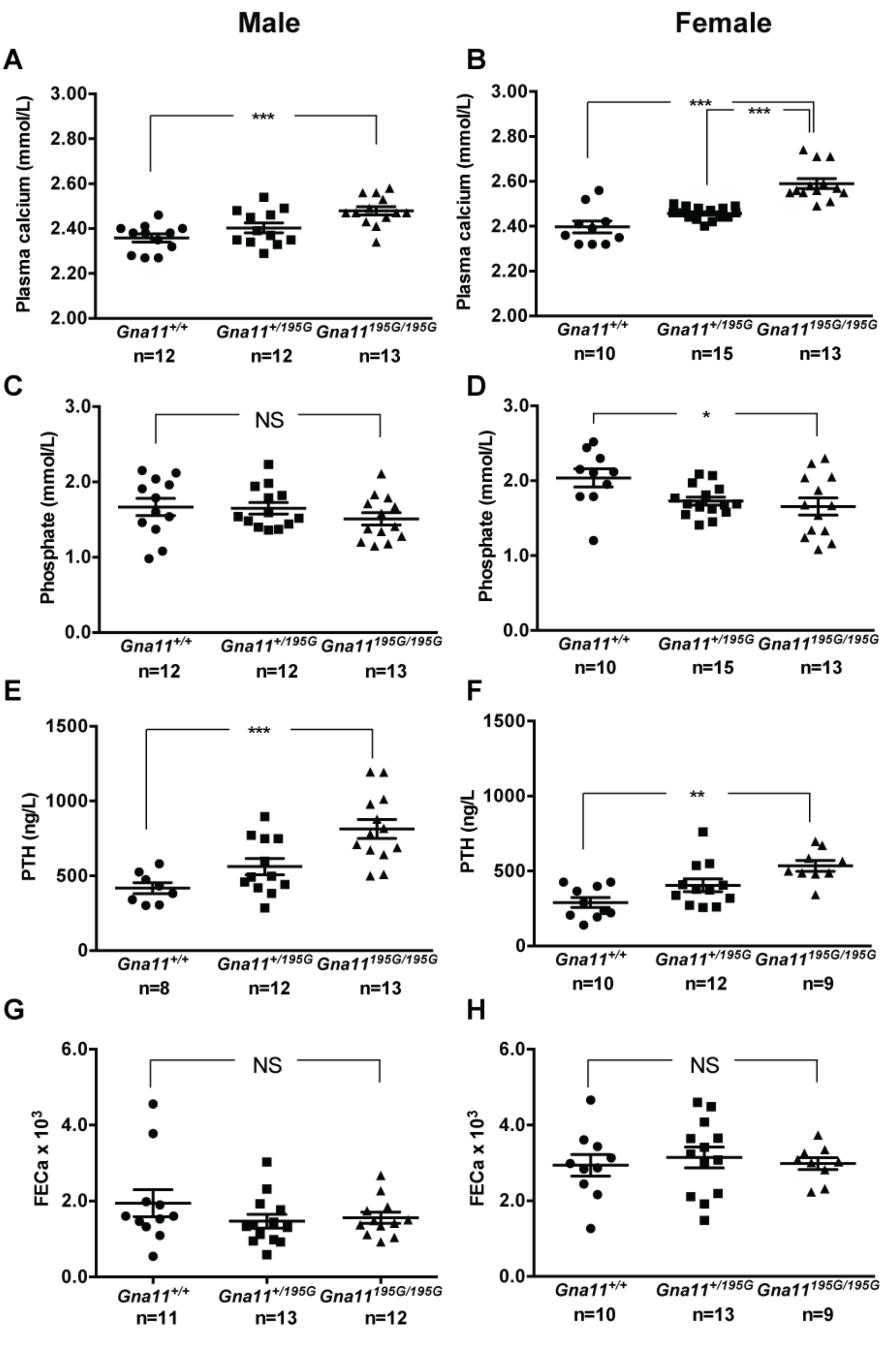

Supplementary Figure 2. Calcitropic phenotype of male and female *Gna11*$^{+/+}$, *Gna11*$^{+/195G}$ and *Gna11*$^{195G/195G}$ mice. (**A-B**) plasma adjusted-calcium concentrations, (**C-D**) plasma phosphate concentrations, (**E-F**) plasma PTH concentrations, and (**G-H**) fractional excretion of calcium (FECa) of male and female *Gna11*$^{+/+}$ (circles), *Gna11*$^{+/195G}$ (squares) and *Gna11*$^{195G/195G}$ (triangles) mice. Mean ± SEM values for the respective groups are indicated by the solid bars. A Kruskal-Wallis test followed by Dunn's test for non-parametric pairwise multiple comparisons were used for analysis of A-H. *p<0.05, **p<0.01, ***p<0.001. NS, non-significant.

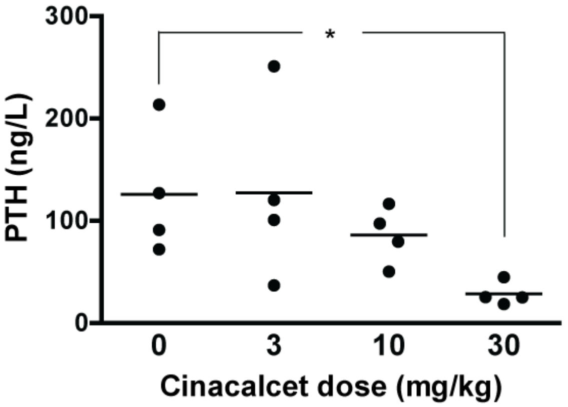

Supplementary Figure 3. Effect of a range of oral cinacalcet doses on plasma PTH concentrations at 2h in WT (*Gna11*$^{+/+}$) mice. Filled circles represent individual WT mice (N=4 mice per dose). Mean±SEM values for the respective groups are indicated by solid bars. Statistical analysis was undertaken using a Kruskal-Wallis test followed by Dunn's test for non-parametric pairwise multiple comparisons. *$p<0.05$.

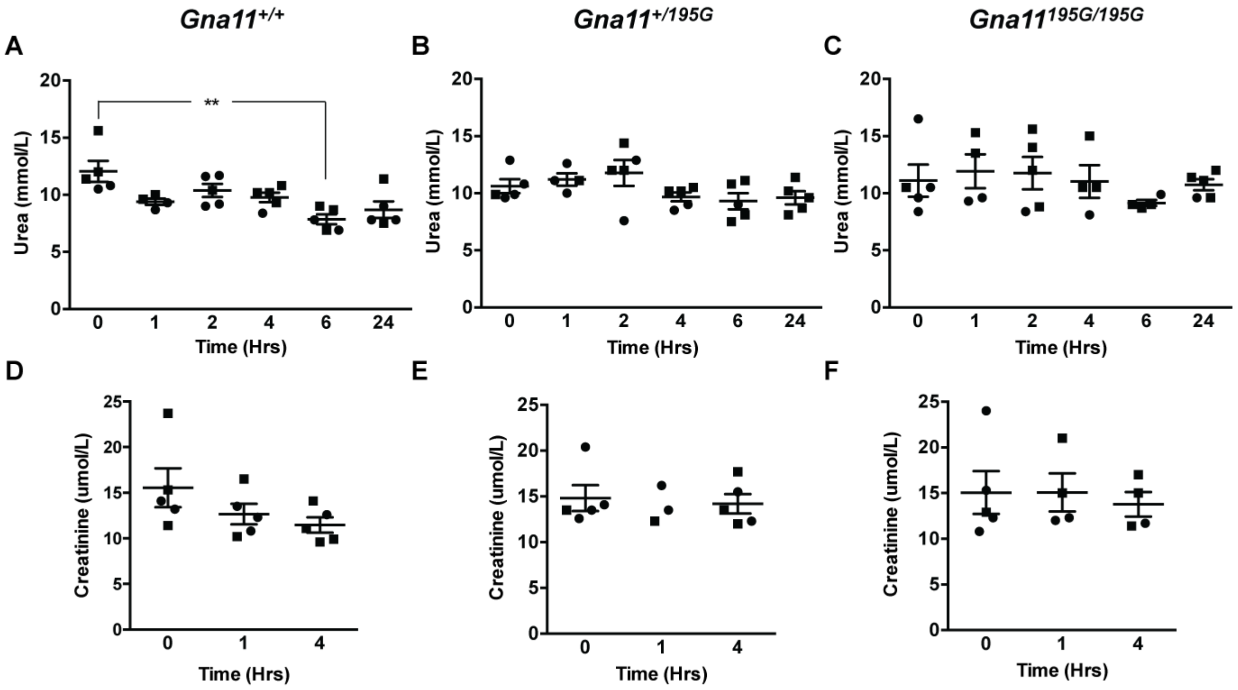

Supplementary Figure 4. Effect of 30 mg/kg cinacalcet on plasma urea and creatinine of *Gna11*$^{+/+}$, *Gna11*$^{+/195G}$ and *Gna11*$^{195G/195G}$ mice. (**A-C**) plasma urea concentrations are shown at 0, 1, 2, 4, 6 and 24h, and (**D-F**) plasma creatinine concentrations are shown at 0, 1 and 4h following oral gavage administration of a single 30mg/kg cinacalcet dose. Mean±SEM values for the respective groups are indicated by solid bars. N=3-5 mice per study time-point. Squares, males; circles, females. A Kruskal-Wallis test followed by Dunn's test for non-parametric pairwise multiple comparisons were used for analysis of A-F. **p<0.01.
